# Tumor Necrosis Factor -α, Interleukin-10, Intercellular and Vascular Adhesion Molecules Are Possible Biomarkers of Disease Severity in Complicated *Plasmodium vivax* Isolates from Pakistan

**DOI:** 10.1371/journal.pone.0081363

**Published:** 2013-12-04

**Authors:** Afsheen Raza, Najia K. Ghanchi, Ali bin Sarwar Zubairi, Ahmed Raheem, Sobia Nizami, Mohammad Asim Beg

**Affiliations:** 1 Department of Pathology and Microbiology, Aga Khan University, Karachi, Sindh, Pakistan; 2 Department of Medicine, Aga Khan Hospital, Karachi, Sindh, Pakistan; 3 Department of Biological and Biomedical Sciences, Aga Khan University, Karachi, Sindh, Pakistan; Centro de Pesquisa Rene Rachou/Fundação Oswaldo Cruz (Fiocruz-Minas), Brazil

## Abstract

**Background:**

Cytokine-mediated endothelial activation pathway is a known mechanism of pathogenesis employed by *Plasmodium falciparum* to induce severe disease symptoms in human host. Though considered benign, complicated cases of *Plasmodium vivax* are being reported worldwide and from Pakistan. It has been hypothesized that *P.vivax* utilizes similar mechanism of pathogenesis, as that of *P.falciparum* for manifestations of severe malaria. Therefore, the main objective of this study was to characterize the role of cytokines and endothelial activation markers in complicated *Plasmodium vivax* isolates from Pakistan.

**Methods and Principle Findings:**

A case control study using plasma samples from well-characterized groups suffering from *P.vivax* infection including uncomplicated cases (n=100), complicated cases (n=82) and healthy controls (n=100) were investigated. Base line levels of Tumor necrosis factor-α (TNF-α), Interleukin-6 (IL-6), Interleukin-10 (IL-10), Intercellular adhesion molecule-1 (ICAM-1), Vascular adhesion molecule-1(VCAM-1) and E-selectin were measured by ELISA. Correlation of cytokines and endothelial activation markers was done using Spearman’s correlation analysis. Furthermore, significance of these biomarkers as indicators of disease severity was also analyzed. The results showed that TNF-α, IL-10, ICAM-1and VCAM-1 were 3-fold, 3.7 fold and 2 fold increased between uncomplicated and complicated cases. Comparison of healthy controls with uncomplicated cases showed no significant difference in TNF-α concentrations while IL-6, IL-10, ICAM-1, VCAM-1 and E-selectin were found to be elevated respectively. In addition, significant positive correlation was observed between TNF-α and IL-10/ ICAM-1, IL-6 and IL-10, ICAM-1 and VCAM-1.A Receiver operating curve (ROC) was generated which showed that TNF-α, IL-10, ICAM-1 and VCAM-1 were the best individual predictors of complicated *P.vivax* malaria.

**Conclusion:**

The results suggest that though endothelial adhesion molecules are inducible by pro-inflammatory cytokine TNF-α, however, cytokine-mediated endothelial activation pathway is not clearly demonstrated as a mechanism of pathogenesis in complicated *P.vivax* malaria cases from Pakistan.

## Introduction

*Plasmodium vivax* is the most common human malarial species causing approximately 216 million clinical infections worldwide [1].Though, highly prevalent, *P.vivax* has been associated with increased morbidity but reduced mortality when compared to its human malarial counterpart *Plasmodium falciparum*. Recently, studies from Indonesia, Papua New Guinea, Brazil, India, Iran and Pakistan have shown that *P.vivax*, either independently or in synergy with co-morbidities/mixed plasmodia species can cause severe or even fatal malaria episodes [2-11]. However, mechanisms associated with *vivax* disease severity have not been characterized to date.

In *P.falciparum*, pathogenesis involves sequestration of parasitized RBCs (pRBCs) to the organ microvasculature via cellular adhesion molecules [12-16]. The expression of these molecules on tissue endothelium is mediated via a process known as the cytokine-mediated endothelial activation pathway [15].Briefly, this pathway is initiated as a result of cell mediated immune response to infectious agents. Recruitment of T cells result in secretion of cytokines on the surface of the endothelial cells. These cells respond by inducing phenotypic changes in the endothelium, leading to expression of specific adhesion molecules, most notably Intercellular adhesion molecule-1(ICAM-1),Vascular cellular adhesion molecule-1(VCAM-1) and E-selectin. These adhesion molecules disturb endothelium permeability and promote leukocyte recruitment and adhesion (of pRBCs and leukocytes).Consequently, transmigration of leukocytes and malaria toxins to the underlying tissues result in injury. Further stimulation and migration of blood monocytes result in the development of macrophages leading to further secretion of inflammatory cytokines and sustaining the stimulus for leukocyte/pRBC adherence, transmigration and tissue injury [17-19].

It has been postulated that *P.vivax* utilizes the same mechanism of pathogenesis to manifest severe disease symptoms. Studies on acute phase uncomplicated *P.vivax* infection have reported raised concentrations of TNF-α, IL-1, IL-4, IL-6, IL-10, IL-12, IFN-γ, ICAM-1,VCAM-1,E-selectin and thrombomodulin [20-30]. However, not all studies have confirmed the relationship between cytokines and soluble CAMs, particularly in well-characterized *P.vivax* complicated cases. Therefore, this study aimed to determine the role of cytokines and endothelial adhesion molecules as possible inducers of cytokine-mediated endothelial activation pathway in complicated *P.vivax* cases from Pakistan. Furthermore, usefulness of respective markers as indicators of disease severity has also been investigated.

## Methods

### Study settings and case definitions

A case control study was conducted between January 2009-December 2011 at the Aga Khan University and Hospital Karachi, Pakistan (AKU) to compare and correlate plasma cytokine levels TNF-α, IL-6, IL-10 and endothelial adhesion molecules ICAM-1, VCAM-1, E-selectin in healthy controls, uncomplicated and complicated P.vivax cases.

#### Case Definitions

Healthy controls: Individuals tested negative on screening test for Hepatitis B, C, Human Immunodeficiency Virus (HIV), syphilis and malaria were recruited as healthy controls.

Uncomplicated malaria (UM): Febrile patients tested slide and PCR positive for *P.vivax* infection but no malarial complication were recruited as uncomplicated cases.

Complicated malaria: Patients tested slide and PCR positive for *P.vivax* infection and admitted to the Aga Khan University Hospital, Karachi with at least one complication per WHO criteria (WHO guidelines) were enrolled as complicated cases [31].

Cases and controls with no co-morbid /associated diagnosis were enrolled in the study. 

### Ethical considerations

The study was approved by the Ethical Review Committee of The Aga Khan University (1811-Pat-ERC-10) and was conducted in accordance with Good Clinical Practice of Declaration of Helsinki [32]. Informed written consent was obtained from all participants. Information regarding co-morbid /associated diagnosis was obtained via questionnaire and medical records.

### Microcopy and PCR

Approximately 2 ml of intravenous blood sample in EDTA tube was collected. Initial presence of malaria parasites was established by Leishman’s staining while further species identification was determined by Giemsa staining of thick and thin blood smears [33]. Plasma was collected by centrifuging remaining blood at 3500 rpm for 15 minutes. Blood and plasma aliquots were stored at -80°C until further analysis.

Complete blood count was performed on automated coulter counter (Beckman Coulter Inc., USA).

DNA was extracted from 200µl of whole blood using QiAamp DNA Mini Kit according to manufacturer’s instructions (Qiagen, USA). Confirmation of *P.vivax* mono-infection was performed using a species-specific PCR [34].

### ELISA for quantification of inflammatory cytokines

TNF-α, IL-6 and IL-10 were detected in plasma of healthy controls and patients by using standards and ELISA reagents obtained from Endogen (Rockford, IL, USA). Cytokines were measured using a sandwich ELISA technique according to the manufacturer's instructions and as reported previously [35]. Recombinant human cytokine was used to obtain a dose response curve with a range of detection from 3.9–1000 pg/ml. All experimental samples were tested in duplicate.

### Elisa for quantification of endothelial adhesion molecules

Quantification of endothelial activation markers ICAM-1, VCAM-1 and E-Selectin was performed using commercially available kits (ICAM-1 and VCAM-1: R&D systems Inc., USA, E-Selectin, Cell Application, Inc., USA). All experimental samples were tested in duplicate according to the manufacturer's recommendations.

A standard curve was constructed for cytokines and endothelial activation markers by plotting mean absorbance for each standard on y-axis against concentration on the x-axis. A best fit curve was plotted through the points on the graph to determine the concentration of the cytokines and endothelial adhesion molecules.

### Statistical analysis

Data was entered in Microsoft Excel and Graph Pad Prism version 5.0 was used for performing further analysis. Receiver Operating Characteristic Curves (ROC) was generated using SAS (Statistical Analysis System) version 9.0. Arithmetical means and medians were calculated, where applicable, for all continuous baseline demographic variables. Kruskal-Wallis test with Dunn’s multiple comparison was used to compare concentrations of cytokines and endothelial adhesion molecules among study groups. Mann-Whitney U test with Bonferroni correction for multiple testing (6 pair wise comparison) was done to verify these differences.

Correlation of cytokines and endothelial adhesion molecules between uncomplicated and complicated cases was performed using Spearman’s Rank correlation analysis. 

Receiver operating characteristic curves (ROC) with C-statistics were used to establish the threshold value of cytokines and endothelial adhesion molecules able to discriminate uncomplicated and complicated *P.vivax* infection. C-statistics determined the Area under curve (AUROC), optimal cutoff with higher likelihood ratio, sensitivity and specificity for each biomarker and validated the ROC curves and the predictive power of each biomarker. Two-tailed p-value <0.05 was considered statistically significant.

## Results

### Baseline demographics, hematological/ clinical presentation of study groups

A total of 220 microscopically confirmed *P.vivax* cases were enrolled in the study. Amongst these, 182 samples tested PCR positive for *P.vivax* mono-infection. Of these, 82 patients had at least one complications as per WHO criteria and were thus identified as complicated cases [31]. 100 patients were recruited as uncomplicated malaria cases while 100 healthy controls were enrolled for the study.

For hematological parameters, thrombocytopenia was a common finding in both complicated and uncomplicated cases. However, profound thrombocytopenia (platelet counts less than 20,000/mm^3^ was observed in complicated cases only. Furthermore, platelet count was approximately 2-fold lower (p-value <0.0001) in complicated cases as compared to uncomplicated cases. Other hematological parameters did not show any significant difference within the study groups.

With respect to clinical complications, pulmonary edema/respiratory distress, metabolic acidosis, hemoglobinuria and jaundice were the common findings in complicated cases.

Baseline demographics, hematological parameters and clinical characteristics of enrolled patients and healthy controls are given in Table 1 and 2.

**Table 1:** Baseline demographics and hematological parameters of the study group.

	**Healthy Controls (HC)**	**Uncomplicated malaria(UM)**	**Complicated malaria(CM)**	**P-value UM vs. CM**
N	100	100	82	
Age (IQR)	38 (25-51)	40 (30-45.2)	44 (34-50)	
Hemoglobin	12.29±1.99	12.38±1.80	12.03±2.14	0.235
RBC	4.38±0.43	4.33±0.55	4.14±0.70	0.073
WBC	5.94±2.43	5.99±2.55	5.94±3.25	0.909
Neutrophils	63.5 ±15	70.5±14.4	68.7±13.7	0.396
Lymphocytes	21.7±11.4	20.1±11.5	21.9±10.8	0.276
Eosinophils	0.97±1.16	0.86±1.19	0.85±0.94	0.930
Monocytes	8.98±5.07	8.12±5.07	8.30±5.02	0.805
Platelets	175±45.78	98.8±42.78	40.2±24.1	*0.0001

Values represent medians ±standard deviation. Age in years, IQR=Interquartile range.

Reference units used: Hemoglobin=gm/dl, RBC=10^12^/L, WBC=x10^6^/L, Platelets=x10^9^/L

Neutrophils, lymphocytes, Eosinophils, Monocytes=%

**Table 2:** Clinical presentations in severe *Plasmodium vivax* infected patients.

**Complications**	**No. of patients**	**% positivity**
Pulmonary edema/Respiratory distress	31	37.8
Metabolic acidosis	20	24.4
Jaundice	26	31.7
Hemoglobinuria	5	6.1

Patients exhibiting at least one symptoms of complicated malaria per WHO criteria.

### Comparison of soluble cytokines and adhesion molecules among study groups

Comparison of plasma concentrations of cytokines between uncomplicated and complicated cases showed that in complicated cases TNF-α and IL-10 were significantly elevated (3-fold and 3.7-fold respectively) while IL-6 was found to be 1.8-fold decreased. Comparison in healthy controls and uncomplicated cases showed no significant difference in TNF-α concentrations while IL-6 and IL-10 was found to be 3.5-fold and 20-fold elevated respectively.

For intercellular adhesion molecules, similar trend was observed. Comparison of uncomplicated and complicated cases showed a significant 2-fold increase in ICAM-1 and VCAM-1 while E-selectin showed 1.2-fold decrease in concentration in complicated cases. Comparison of healthy controls with uncomplicated cases showed significant increase, approximately 3-fold, 4-fold and 10-fold in ICAM-1, VCAM-1 and E-selectin levels.

**Figure 1:**
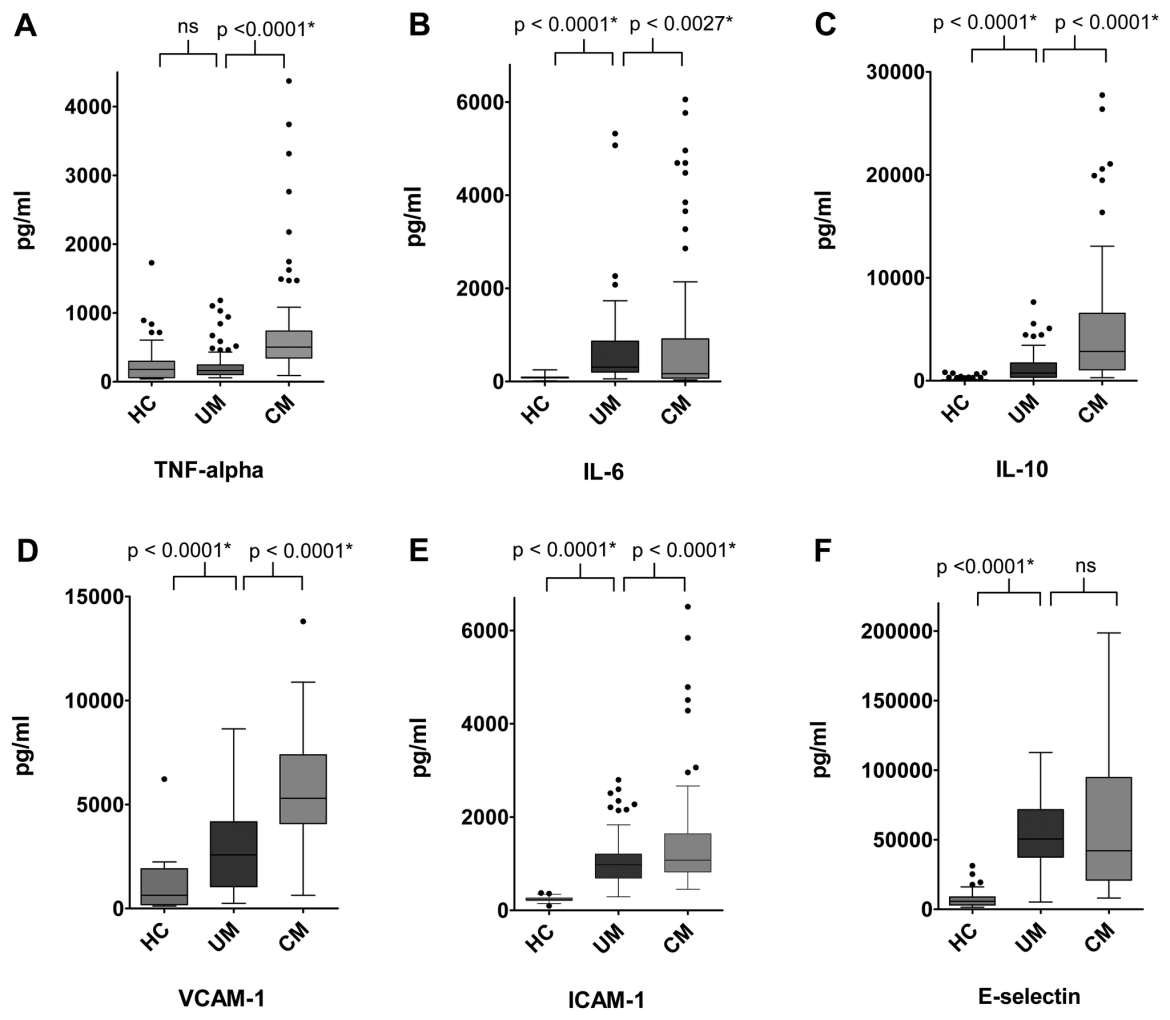
Graphs showing medians with ranges of A) TNF-α B) IL-6 C) IL-10 D) VCAM-1 E)ICAM-1 and F) E-selectin levels in healthy controls, uncomplicated and complicated cases as measured by ELISA (Mann-Whitney). ns=not-significant, p<0.05 after Bonferroni correction for multiple comparisons (6 pair wise comparisons).Healthy controls N=100,Uncomplicated malaria N=100, Complicated malaria N=82.

Comparison of cytokine and endothelial adhesion molecules within groups is given in Figure 1.

### Correlation of cytokines and endothelial adhesion molecules between uncomplicated and complicated cases

Spearman’s Rank correlation analysis was performed in uncomplicated and complicated cases to determine whether complex interactions occurred between cytokines and endothelial adhesion molecules.

In uncomplicated cases, a highly significant negative correlation in TNF-α and VCAM-1 (r= -0.261, p-value<0.009) while a significant positive correlation between IL-6 and IL-10 (r= 0.210, p-value<0.03) was observed.

In complicated cases, significant positive correlation was observed between TNF-α and ICAM-1 (r= 0.314, p-value=0.004), TNF-α and IL-10 (r= 0.287, p-value=0.009), IL-10 and IL-6 (r=0.345, p-value=0.002) and ICAM-1 and VCAM-1(r= 0.237, p-value<0.03) (Table 3).

**Table 3:** Correlation between cytokines and endothelial adhesion molecules in uncomplicated and complicated *P.vivax* cases.

	**TNF-α**	**IL-6**	**IL-10**	**ICAM-1**	**VCAM-1**	**E-Selectin**
**Uncomplicated malaria**						
TNF-α	1.00	-0.099	0.049	-0.071	-0.261**	0.031
IL-6		1.00	0.210^*^	-0.125	-0.130	-0.054
IL-10			1.00	-0.005	0.108	-0.064
ICAM-1				1.00	0.413	0.008
VCAM-1					1.00	-0.082
E-selectin						1.00
**Complicated malaria**						
TNF-α	1.00	-0.089	0.287^**^	0.314^**^	-0.065	0.048
IL-6		1.00	0.345**	-0.126	-0.059	-0.215
IL-10			1.00	0.130	-0.082	0.162
ICAM-1				1.00	0.237^*^	0.065
VCAM-1					1.00	0.008
E-selectin						1.00

Spearman Rank Correlation analysis was carried out between cytokines and endothelial adhesion molecules in uncomplicated and complicated cases. Correlation coefficient (rho) is given. Correlation is significant at p- value <0.05.*denotes significant results, **denotes highly significant results.

### Cytokines and endothelial adhesion molecules as biomarkers of disease severity

**Figure 2:**
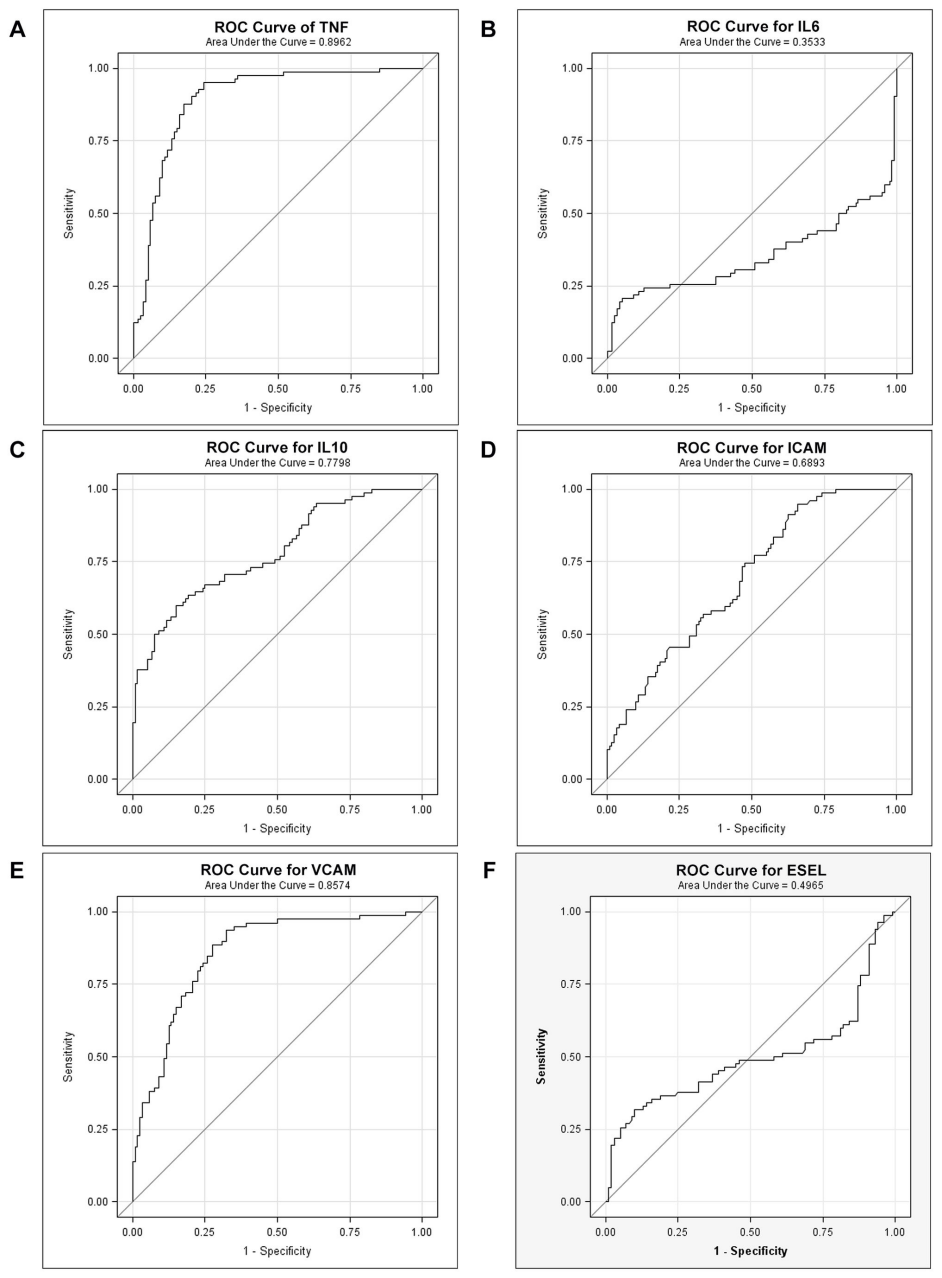
a, b, c: Effectiveness of cytokines, TNF-α, IL-6 and IL-10 measurements as markers of disease severity using Receiver operating curve (ROC); d, e, f: Effectiveness of endothelial activation markers, ICAM-1, VCAM-1 and E-selectin as markers of disease severity using Receiver operating curve (ROC). Solid straight line represents predictive threshold while solid curves line represents the sensitivity and specificity of the respective cytokine and endothelial activation markers. A threshold of 0.5 was used for this study.

To determine whether cytokines and endothelial adhesion molecules could be informative in differentiating complicated *P.vivax* cases from uncomplicated cases, a Receiver Operating Characteristic (ROC) curve was generated to determine diagnostic accuracy of these markers. It was observed that TNF-α, IL-10, ICAM-1 and VCAM-1 were the 4 best individual predictors of complicated vivax malaria with AUROC of 0.89 (95% CI: 0.84 - 0.94), 0.79 (95%CI: 0.73- to 0.86), 0.63 (0.55-0.71) and 0.84 (95% CI: 0.78- 0.90) respectively. The sensitivity and specificity of these molecules was between 91-99% and 75-82% respectively (Table 4; Figure 2a-2f).

**Table 4:** Receiver Operating Characteristic (ROC) curves of cytokines and endothelial biomarkers in uncomplicated vs. complicated vivax malaria cases.

	**AUROC UM vs. CM (95% CI)**	**p-value**	**Cutoff**	**Sensitivity% (95% CI)**	**Specificity % (95% CI**)	**Likelihood ratio**
**TNF-α**	0.89(0.8- 0.94)	< 0.0001	>91.40	98.8 (93-99)	82(73-89)	1.20
**IL-6**	0.63(0.54-0.72)	<0.003	<403.6	70(58-79 )	62(52-72)	1.12
**IL-10**	0.79(0.73- 0.86)	< 0.0001	> 312.5	99(93- 99)	79(69-86)	1.25
**ICAM-1**	0.63(0.55-0.71)	<0.004	> 694.4	91.5(83-97)	75(65-83)	1.22
**VCAM-1**	0.84(0.78-0.90)	< 0.0001	>930.5	99(93-99.9)	80(70-87)	1.23
**E-selectin**	0.50(0.41- 0.60)	0.94	< 47534	50(40-60)	51(40-62)	1.02

AUROC: Area Under Curve, CI: Confidence Interval

## Discussion

Cytokines and endothelial adhesion molecules were correlated in complicated *Plasmodium vivax* malaria cases from Pakistan to investigate the possible role of these molecules in manifestation of severe disease. Furthermore, biomarkers as predictors of disease severity in complicated malaria are also described.

Plasma concentrations of cytokines, TNF-α, IL-6, IL-10 and endothelial adhesion molecules, ICAM-1, VCAM-1and E-selectin were compared among healthy controls, uncomplicated and complicated cases. In TNF-α, significant increase was observed between uncomplicated and complicated cases; however, no significant difference was observed in uncomplicated cases and healthy controls. This finding is consistent with previously reported observations [20,27,29] and implies a similar immunological response in patients suffering from malaria. Excessive up regulation of TNF-α observed in complicated cases indicates host response to contain the infection and prevent tissue injury. However, this dysregulated expression of TNF-α may possibly lead to tissue injury via over expression of endothelial adhesion molecules and disturbance in the endothelium permeability [36,37].

A sequential increase in IL-10 concentration was observed among healthy controls, uncomplicated and complicated cases. Previous studies have demonstrated that severe *P.vivax* malaria exhibits inflammatory imbalance with predominance of pro-inflammatory response [27]. Other studies have documented no clear association of pro-inflammatory imbalance with disease severity [29]. Data from our study demonstrate similar results. Regulatory cytokines, such as IL-10 are required to reduce the risk of severe disease or more tissue injury. Some studies on *P.falciparum* malaria have reported that high IL-10 levels may sometimes aggravate disease severity by inhibiting parasite killing effector mechanisms [38].However; our study did not focus on effector molecules and thus, does not provide evidence on IL-10 and host injury. Therefore, the role of IL-10 and tissue injury cannot be commented upon.

IL-6 levels were found to be elevated in healthy controls and uncomplicated cases. However, comparison of IL-6 levels between uncomplicated and complicated cases showed a significant decrease in complicated cases. This finding is distinct from earlier reports which documented no significant difference in IL-6 expression between symptomatic and asymptomatic *P.vivax* study subjects [23,29]. IL-6 is a cytokine that acts either as a pro-inflammatory or anti-inflammatory cytokine depending upon the stimulus. It is possible that *P.vivax* isolates circulating in Pakistan may be modulating the immune response to trigger IL-6 pro-inflammatory response. During complicated infection, overly expressed TNF-α and IL-10 thus act to downregulates IL-6 production. However further studies from Pakistan on cytokine expression in response to *P.vivax* infection are needed to corroborate these findings.

Comparison of three inducible adhesion molecules in uncomplicated and complicated cases showed that ICAM-1 and VCAM-1 levels were significantly higher (p value<0.0001) while E-selectin levels were found to be lower in complicated cases. The expression of ICAM-1 and VCAM-1 is consistent with previously reported data indicating these adhesion molecules are expressed during complicated malaria [25,26,37,39-41]. However, decreased expression of E-selectin in complicated cases is a distinct finding and may be due to two reasons: firstly, expression of E-selectin in most organs is transient and is induced by *de novo* synthesis on endothelium, peaking within 2 - 6 h in response to inflammatory stimuli and subsiding to basal levels within 10 - 24 hours [19]. Therefore, it is possible that the timing of sample collection may have a critical influence on the result. Secondly, E-selectin expression is known to be rapidly down regulated during infection to protect the host against harmful effects of inflammation [19]. It is possible that decrease in E-selectin concentration in complicated cases is observed due to this reason. However, these are preliminary conclusions which need validation by further studies on E-selectin expression in *P.vivax*.

To determine whether complex interactions occur between cytokines and endothelial adhesion molecules during disease state, Spearman correlation analysis was performed. Uncomplicated cases showed a positive correlation for IL-6 and IL-10 whilst TNF-α and VCAM-1 showed a negative correlation. As shown in Figure 1, there was no difference in TNF-α level while increased VCAM-1 levels were observed in healthy and uncomplicated cases, thus corroborating this negative correlation. However, positive correlation between IL-6 and IL-10 level (with increase in concentration observed in Figure 1) may possibly be involved in expression of VCAM-1. Interestingly, in complicated cases, significant positive correlation was observed in TNF-α and ICAM-1 indicating that high levels of TNF-α together with IL-10, act on vascular endothelium to up regulate expression of other adhesion molecules, especially ICAM-1. Furthermore, correlation of ICAM-1 and VCAM-1 indicates interaction of both these molecules on the endothelium in response to inflammatory stimuli. The effect of this interaction may play a possible role in pRBC, platelet and leukocyte adherence to microvasculature of organs. In addition, possible disturbance to permeability of the endothelium may be triggered, resulting in transmigration of leukocyte and malaria toxins to the underlying organs. Studies in severe *P.falciparum* infection have reported similar observations [40] thus corroborating findings in this study. In complicated cases, correlation between IL-6 and IL-10 is a distinctive finding (as IL-6 expression was found to be decreased in complicated cases in this study) and requires extensive studies to understand the dynamic interaction of respective cytokines in complicated *P.vivax* infection.

The most common clinical complication observed in our study was pulmonary edema (observed in 37.8% of the patients). This finding is consistent with previously reported data from Pakistan, India, Indonesia, Papua New Guinea, and Brazil [2,4,7,10,11,41-43] indicating that lung is an organ most commonly affected during complicated *P.vivax* infection. It has been hypothesized and reported that lung dysfunction in *P.vivax* may be a consequence of excessive intravascular inflammatory response and endothelial adhesion molecules expression such as CD36, ICAM-1 etc that leads to changes in endothelium permeability, transmigration of leukocytes and accumulation of interstitial/alveolar edema [4,24,44-46]. Though, elevated levels of cytokines and endothelial adhesion molecules are observed in patients suffering from pulmonary edema/respiratory distress in our study, however, no clear cut conclusion on the role of these molecules can be drawn based on only 31 patients.

Thrombocytopenia was found to be significantly disturbed in both uncomplicated and complicated cases though profound thrombocytopenia (platelets less than 20,000/mm^3^) was observed only in complicated cases. Thrombocytopenia in malaria is reported to be a consequence of anti-body induced platelet destruction, coagulopathy and platelet adhesion to the activated endothelium [47, 48]. In *P.falciparum*, these adherent platelets serve as a bridge to facilitate pRBC binding to the endothelial adhesion molecules [49, 50]. In *P.vivax*, thrombocytopenia due to platelet consumption via endothelial adherence/ destruction and coagulopathy has been documented [28]. Findings from our study, of profound thrombocytopenia, cytokine and endothelial adhesion molecule expression imply a similar trend in *P.vivax* malaria in Pakistan. However, with regard to thrombocytopenia, assumptions and not conclusions can be drawn from our findings since this study investigated a generalized mechanism in complicated vivax malaria rather than specific platelet activated molecules.

Previous studies have reported the plausibility of cytokines and endothelial adhesion molecules as markers to differentiate uncomplicated and complicated malaria infection [20,27,40]. Therefore, the respective molecules as biomarkers of *P.vivax* disease severity were evaluated using ROC curves. Interestingly, ROC curve generated indicated that TNF-α, IL-10, ICAM-1 and VCAM-1 were the best individual predictors of complicated *P.vivax* malaria. Previous studies on *P.vivax* have documented similar results for TNF-α and IL-10 [20,27] while results for ICAM-1 and VCAM-1 are consistent with that reported for complicated *P.falciparum* infection [40]. Endothelial adhesion molecules ICAM-1 and VCAM-1 have not been described as biomarkers for differentiation of complicated cases in *P.vivax* and this is the first study to investigate this aspect. Therefore, further studies from Pakistan on complicated *P.vivax* infection, are suggested in order to validate the use of these biomarkers in prognosis and treatment assessment.

Previous studies have suggested that parasite biomass influences endothelial activation and production of both pro- and anti-inflammatory cytokines [23]. However, information on parasitaemia was not considered in this study which is a limitation of our study. It is suggested that further studies focusing on this aspect should be conducted from Pakistan to understand the effect of this factor on cytokine and endothelial molecule expression. 

## Conclusion

This is the first study from Pakistan to determine and correlate baseline levels of cytokines and endothelial adhesion molecules in *P.vivax* infected patients and determine the plausibility of these biomarkers as indicators of disease severity in complicated cases. The results suggest that though endothelial adhesion molecules are inducible by pro-inflammatory cytokine TNF-α, however, cytokine-mediated endothelial activation pathway is not clearly demonstrated as a mechanism of pathogenesis in complicated *P.vivax* malaria cases.
